# Spatio-temporal analysis of malaria incidence at the village level in a malaria-endemic area in Hainan, China

**DOI:** 10.1186/1475-2875-10-88

**Published:** 2011-04-14

**Authors:** Liang Wen, Chengyi Li, Minghe Lin, Zhengquan Yuan, Donghui Huo, Shenlong Li, Yong Wang, Chenyi Chu, Ruizhong Jia, Hongbin Song

**Affiliations:** 1 PLA Institute of Disease Control and Prevention, Beijing, China; 2 Wanning Health and Epidemic Prevention Station, Wanning County, Hainan province, China

## Abstract

**Background:**

Malaria incidence in China's Hainan province has dropped significantly, since Malaria Programme of China Global Fund Round 1 was launched. To lay a foundation for further studies to evaluate the efficacy of Malaria Programme and to help with public health planning and resource allocation in the future, the temporal and spatial variations of malaria epidemic are analysed and areas and seasons with a higher risk are identified at a fine geographic scale within a malaria endemic county in Hainan.

**Methods:**

Malaria cases among the residents in each of 37 villages within hyper-endemic areas of Wanning county in southeast Hainan from 2005 to 2009 were geo-coded at village level based on residence once the patients were diagnosed. Based on data so obtained, purely temporal, purely spatial and space-time scan statistics and geographic information systems (GIS) were employed to identify clusters of time, space and space-time with elevated proportions of malaria cases.

**Results:**

Purely temporal scan statistics suggested clusters in 2005,2006 and 2007 and no cluster in 2008 and 2009. Purely spatial clustering analyses pinpointed the most likely cluster as including three villages in 2005 and 2006 respectively, sixteen villages in 2007, nine villages in 2008, and five villages in 2009, and the south area of Nanqiao town as the most likely to have a significantly high occurrence of malaria. The space-time clustering analysis found the most likely cluster as including three villages in the south of Nanqiao town with a time frame from January 2005 to May 2007.

**Conclusions:**

Even in a small traditional malaria endemic area, malaria incidence has a significant spatial and temporal heterogeneity on the finer spatial and temporal scales. The scan statistics enable the description of this spatiotemporal heterogeneity, helping with clarifying the epidemiology of malaria and prioritizing the resource assignment and investigation of malaria on a finer geographical scale in endemic areas.

## Background

Malaria is widespread in tropical and subtropical regions. It was estimated that about 3.3 billion people were at risk of malaria in 2006[1]. In 2008, there were about 243 million malaria cases worldwide, which accounted for an estimated 863,000 deaths[2]. Malaria is also a leading parasitic diseases in China as in early 1970's, China's malaria cases hit 24 million [3]. With nationwide efforts made, regions of high exposure to severe malaria epidemics have been downsized, e.g., cases reported in China in 2008 fell to 26,873[4].

At the beginning of 2006, the National Malaria Control Programme 2006-2015 was formulated by the Ministry of Health of China. It aims at blocking malaria transmission both in all hyper-endemic border counties in Yunnan province, and in central-southern mountainous areas of Hainan province by the end of 2015. In addition, the programme is designed to eliminate malaria in this timeframe in other endemic areas and *Plasmodium falciparum *malaria in Hainan Province[3].

Hainan, the second largest island and the smallest land province in China, lies in the South China Sea, overlooking the mainland to the north across the Qiongzhou Strait. Tropical monsoon and tropical marine climate feature high temperatures and rich rainfall, an ideal environment for malaria transmission. Hainan province is characteristic of the highest malaria incidence in China for many years, as one of the only two provinces with *P. falciparum *malaria [3].

Malaria Programme of China Global Fund Round 1 implemented in Hainan from April 2003 to March 2008 is designed to enable timely diagnosis, standard treatment, effective prevention, health education and promotion of malaria. In addition, it aims to strengthen malaria surveillance and project management capabilities for curbing malaria epidemic and the spread of multi-resistant *P. falciparum *malaria. Malaria Programme of Round 5, implemented in Hainan since 2006, aims to further control malaria epidemic and eliminate malaria in the end by consolidating the results achieved since Round 1. The Programme has achieved downsizing of malaria cases in Hainan as the incidence has fallen to the second in China in 2006 [5], and to the third in 2008 [4].

As the risk of *Plasmodium *infection varies with space and time, a precise knowledge of the regions at risk, the level of risk, risk factors and the exposed population is needed before taking local-specific anti-malaria actions. WHO recommends the stratification of malaria risk. This calls for an analysis of local variations, e.g., define high-risk zones on a finer geographical scale, for the purpose of taking more efficient anti-malaria measures [6].

Recent years have reported a few spatiotemporal research of malaria in China, yet mostly limited to county level[7-11], which confines the knowledge of malaria distribution to a general picture. This study aims to reveal the temporal and spatial variation of malaria transmission in a hyper-endemic area in Hainan between 2005 and 2009 at village level, to pave the way for further studies such as evaluating the efficacy of Malaria Programme of Round 1 and Round 5 and identifying the environmental and landscape characteristics associated with increased risk for malaria infections on a finer geographic scale.

## Methods

### Study area

**Figure 1:**
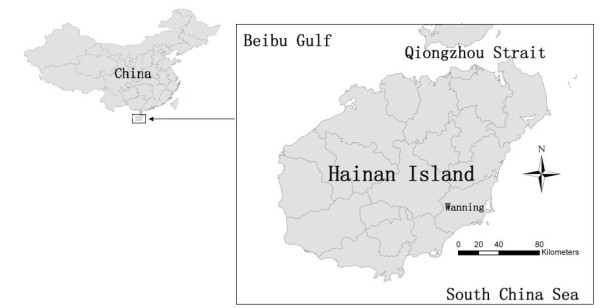
**Location of Wanning county on Hainan island, China**.

Wanning County resides at the south-east of the Hainan Island (Figure 1) with a tropical monsoon climate featuring ample sunshine and precipitation. Its average temperature of the coldest month and the hottest month are 18.7°C and 28.5°C respectively. The west of Wanning is mountainous area, central part is hilly land, and south-east seacoast is plain. During the five-year study (2005-2009), the population increased from 0.56 million to 0.6 million.

**Figure 2:**
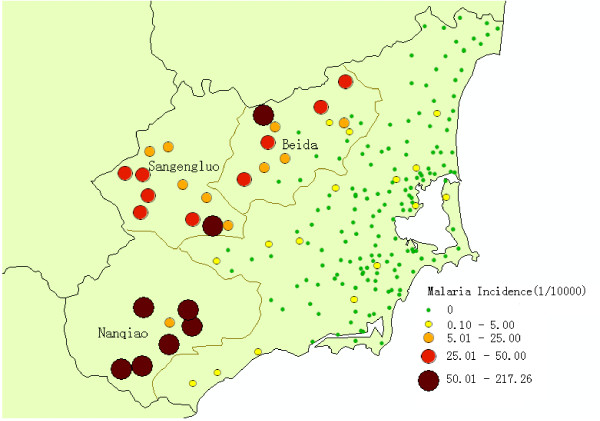
**Annual malaria incidence of all 197 villages in Wanning county from 2005 to 2009**.

Wanning is one of the traditional malaria endemic area and the malaria cases have dropped significantly since it participated in Malaria Programme of China Global Fund Round 1 in 2003. 963 malaria cases were reported in its 197 administrative villages from 2005 to 2009. Especially noteworthy is that 942(97.82%) cases of which were found in 29 administrative villages in Nanqiao town, Sangengluo town and Beida town at the western mountainous areas, while no or few cases were reported in the central and coastal villages (Figure 2). The study covered all the 37 administrative villages in Nanqiao, Sangengluo and Beida, with a population increased from 40,766 to 42,614 during 2005 and 2009.

### Data sources

Records on malaria cases and the number of residents in each village in every year from 2005 to 2009 were provided by the Health and Epidemic Prevention Station of Wanning County, an organization responsible for national notifiable communicable disease surveillance and report in the county. The malaria cases included both microscopically confirmed and clinically diagnosed cases. Of those, the clinically diagnosed ones fit to the definition of clinically diagnosed malaria case in Diagnostic Criteria and Principles of Management of Malaria of China's national standard (GB 15989--1995). Not all of these cases were further confirmed microscopically, because not all the basic health facilities have this capability in many malaria endemic regions in China. In China, the clinical cases without microscopically diagnosed are also taken into account in calculation of the total incidence of malaria. The digital map for a GIS-based analysis on the spatial distribution of malaria has the point layer with information on the latitudes and longitudes of central points of each village. The malaria cases and incidence of each village were matched to the village-level layer of the point by village code using the software ArcGIS 9.1 (ESRI Inc., Redlands, CA, USA).

### Cluster analysis

Scan statistics were used to detect and evaluate the clusters of cases in either a purely temporal, purely spatial or space-time setting. This was done by gradually scanning a window across time and/or space, noting the number of observed and expected observations inside the window at each location. For each location and size of the scanning window, the alternative hypothesis was that there was an elevated risk within the window as compared to outside.

In SaTScan software[12], the scanning window was an interval (in time), a circle or an ellipse (in space) or a cylinder with a circular or elliptic base (in space-time). Multiple different window sizes were used. The window with the maximum likelihood was the most likely cluster, that was, the cluster least likely to be due to chance. A p-value was assigned to this cluster. The standard purely spatial scan statistic imposed a circular window on the map. The window was in turn centered on each of several possible grid points positioned throughout the study region. For each grid point, the radius of the window varied continuously in size from zero to some upper limit specified by the user. In this way, the circular window was flexible both in its location and size, while each circle was a candidate cluster. The space-time scan statistic was defined by a cylindrical window with a circular (or elliptic) geographic base and with its height corresponding to the time. The base was defined exactly as for the purely spatial scan statistic, while the height reflected the time period of potential clusters. The cylindrical window was then moved in space and time, so that for each possible geographical location and size, it also visited each possible time period. In effect, an infinite number of overlapping cylinders of different sizes and shapes were obtained, which jointly cover the entire studied region, where each cylinder reflected a possible cluster. The temporal scan statistic used a window that moved in one dimension, time, defined in the same way as the height of the cylinder used by the space-time scan statistic. This meant that it was flexible in both start and end date.

For purely spatial and space-time analyses, SaTScan also identified secondary clusters in the data set in addition to the most likely cluster, and lined them up by their likelihood ratio test statistic. For purely temporal analyses, only the most likely cluster was reported.

No geographic overlap was used as a default setting, so secondary clusters would not overlap the most significant cluster. In order to scan from small to large clusters, the maximum cluster size was set to 50% of the total population at risk. To ensure sufficient statistical power, the number of Monte Carlo replications was set to 999.

## Results

### Space and time distribution of cases from 2005-2009

A total of 942 malaria cases were reported from 2005 to 2009 in 37 villages. Eight villages of which covering 21.02% of the total population reported no case, and all of them were located at Beida. Eight villages covering 26.31% of the total population had malaria incidence higher than 5‰ and six of them were located at Nanqiao, while the other two located at Sangengluo and Beida respectively (Figure 2). The highest annualized average incidence at village-level was 21.73‰.

**Figure 3:**
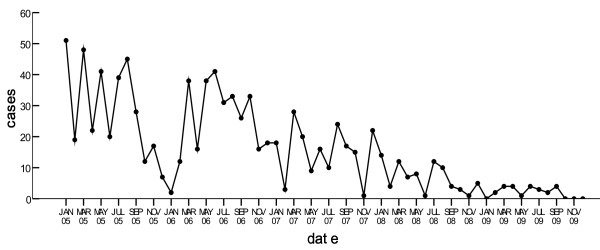
**Monthly reported number of malaria cases in 37 villages in Wanning county from 2005 to 2009**.

**Figure 4:**
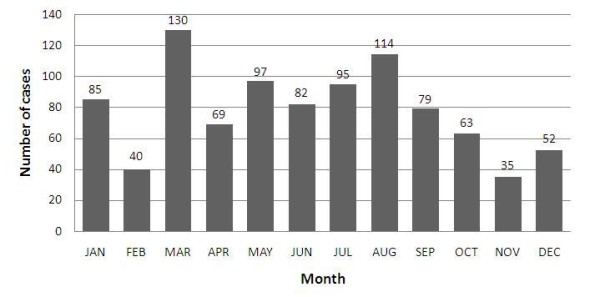
**Total reported number of malaria cases by month in 37 villages in Wanning county from 2005 to 2009**.

Steady drop of infection was obvious. From 2005 to 2009, the cases reported on yearly basis were 349, 304, 184, 81 and 24 respectively. Except four months in 2009, malaria cases were reported each month during the five years, and the largest number of cases of 51 was reported in January 2005(Figure 3). The month with the lowest malaria cases was November during the five years, and that with the largest cases was March (Figure 4).

### Purely temporal clustering

The results of the purely temporal clustering analysis of malaria data by year from 2005 to 2009 were shown in Tables 1. The temporal clusters of malaria cases in the study area included six months in 2005(March to August), six months in 2006(May to October), two months in 2007(March and April) respectively, which in the end disappeared in 2008 (LLR = 3.22, P > 0.05 (and 2009 (LLR = 3.24, P > 0.05).

**Table 1:** The clusters of malaria cases detected using the purely temporal analysis

Year	Cluster time frame	**Obs**^**a**^	**Exp**^**b**^	Relative risk	**LLR**^**c**^	P
2005	2005/3/1 - 2005/8/31	215	175.93	1.58	8.83	0.00
2006	2006/5/1 - 2006/10/31	202	153.25	1.95	15.95	0.00
2007	2007/3/1 - 2007/4/30	48	30.58	1.77	5.25	0.02
2008	2008/1/1 - 2008/5/31	45	33.64	1.76	3.22	0.11
2009	2009/2/1 - 2009/7/31	18	11.90	3.05	3.24	0.12

a: The number of observed cases in a cluster; b: The number of expected cases in a cluster;

c: Log likelihood ratio

### Purely spatial clustering

**Figure 5:**
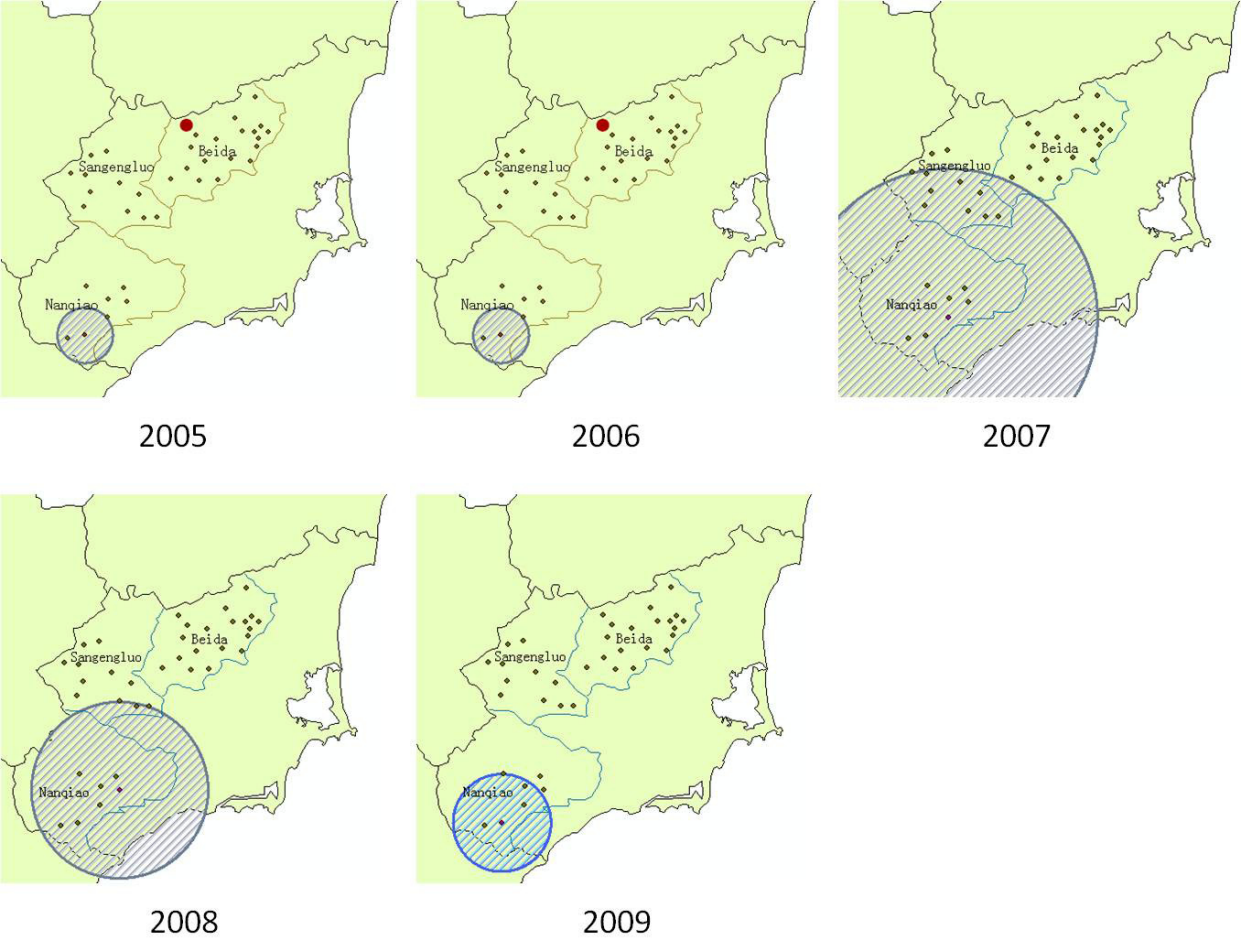
**Locations of the detected clusters of malaria cases from 2005 to 2009, based on the purely spatial analysis**.

Analysis of purely spatial clustering of malaria cases from 2005 to 2009 showed that malaria was not distributed randomly in the study area (Table 2 and Figure 5). With the maximum spatial cluster size of 50% of the total population, the spatial clustering analysis identified the most likely cluster for each of the five years. The cluster covered three villages at Nanqiao in 2005; three villages the same as those of 2005 in 2006; 16 villages of which seven villages at Nanqiao and nine villages at Sangengluo in 2007; nine villages of which seven villages at Nanqiao and two villages at Sangengluo in 2008; and all five villages from Nanqiao in 2009. The secondary cluster was also detected respectively in 2005 and 2006, which covered the same village at Beida. These results showed that Nanqiao was most likely to have a significantly high incidence of malaria.

**Table 2:** The clusters of malaria cases detected using the purely spatial analysis

		Cluster			**Obs**^**a**^	**Exp**^**b**^	Relative risk	**LLR**^**c**^	P
					
Year		Latitude(N)	Longitude(E)	Radius(Km)					
2005	A	18.64666	110.10916	4.72	176	47.36	6.50	134.99	0.00
	B	18.96730	110.26496	0	31	5.48	6.11	29.17	0.00
2006	A	18.64666	110.10916	4.72	161	41.41	7.14	131.70	0.00
	B	18.96730	110.26496	0	34	4.84	7.79	38.60	0.00
2007	A	18.67405	110.14340	25.11	167	86.37	11.68	81.34	0.00
2008	A	18.69753	110.17359	15.18	71	26.41	14.67	53.24	0.00
2009	A	18.64666	110.10916	8.37	18	4.94	11.59	16.35	0.00

a: The number of observed cases in a cluster; b: The number of expected cases in a cluster;

c:Log likelihood ratio; A: Most likely cluster; B: Secondary cluster.

### Space-time clustering

The space-time clustering analysis of the malaria data from 2005 to 2009 was tested. Table 3 shows the most likely cluster for a high incidence of malaria, and depicts it on the map in Figure 6. The most likely cluster had all three villages at Nanqiao (13.58% of the study area's total population) and the time frame was from January 2005 to May 2007. Two secondary clusters were also detected. One of which covers one village at Beida for the time frame from February 2005 to February 2007, while the other had 16 villages, of which four at Nanqiao, eleven at Sangengluo, one at Beida, for the time frame from January 2005 to March 2005.

**Table 3:** The clusters of malaria cases detected using the space-time analysis

Clusters	Time frame	Coordinates/radius	**Obs**^**a**^	**Exp**^**b**^	**RR**^**#**^	**LLR**^**c**^	*p*
A	05/1/1-- 07/5/31	(18.65 N, 110.11 E)/4.72 km	374	60.91	9.53	429..44	0.00
B_1	05/2/1-- 07/2/28	(18.97 N, 110.26 E)/0 km*	70	6.10	12.33	109.20	0.00
B_2	05/1/1-- 05/3/31	(18.84 N, 110.11 E)/17.68 km	59	19.10	3.23	27.52	0.00

A: Most likely cluster; B: Secondary cluster; a: The number of observed cases in a cluster;

b: The number of expected cases in a cluster; c: Log likelihood ratio;

#: Relative risk. *: Only 1 village was included in the cluster;

**Figure 6:**
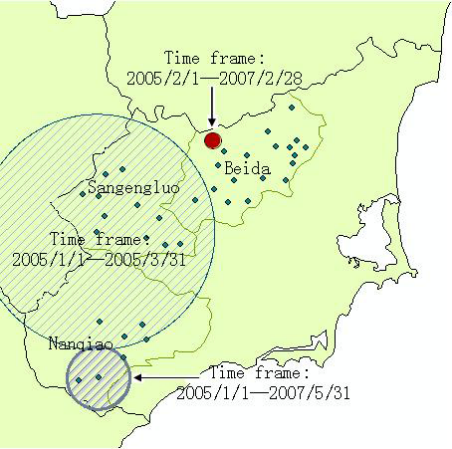
**Locations of the detected clusters of malaria cases from 2005 to 2009, based on the space-time analysis**.

## Discussion

Local factors linked to the malaria transmission must be identified before initiating control programs or studies. Specific management of an environment favouring the proliferation of vectors can significantly decrease the transmission, while intervention alternatives and their relative importance rely on the understanding of environmental heterogeneity at a sufficiently fine-scale[6]. For that, the pure temporal, pure spatial and spatio-temporal cluster analyses were conducted to explore the temporal and spatial variations of malaria at village level within a traditional malaria hyper-endemic area in Hainan, China, from 2005 to 2009.

Purely temporal clustering in each year could indicate the seasonal tendency of the malaria transmission while pure spatial clustering could find out high-risk areas over the large region. These results are conducive to more targeted control actions. Space-time clustering indicates the spatial and temporal variations of tendency in a long time period, which could evaluate the effects of control measures. Although the whole study area is traditionally classified as a hyper-endemic of malaria, the identification of clusters demonstrated a high variability of malaria risk over space and time.

Purely temporal clustering indicated that there were high-risk months of malaria in each year between 2005 and 2007. The temporal cluster in 2006 was consistent with the epidemic season of malaria in Hainan province, but those of 2005 and 2007 were found before the epidemic season. The epidemic season of malaria usually falls within the rainy season in Hainan[13,14]. As the meteorological parameters have close links with the variation of malaria incidence, they could be utilized to effectively predict the incidence of malaria province-wide in Hainan, and the whole nine endemic counties at central-south of Hainan Island [13,15]. But on such a finer geographical scale as hyper-endemic area in Wanning, climate impacts on the malaria transmission still need to be identified. Significant temporal clustering disappeared in 2008 and 2009, which might result from a significant reduction of malaria cases thanks to the prevention and control efforts offsetting the impacts caused by meteorological factors.

Purely spatial clustering identified high-risk areas in each year, which showed the complexity of the spatial distribution of malaria within the traditional malaria hyper-endemic area of Wanning. The space distribution of malaria is closely related to the topography of the environment, and the incidence of malaria is lower in most of the villages at flat and open areas than those near foothills in Hainan province[14]. Spatial cluster analysis identified that Nanqiao, especially in its south, as high-risk areas between 2005 and 2009. Because *Anopheles dirus *is the principal vector of malaria at mountainous area in Hainan province[16], it is believed that the epidemics of malaria in Nanqiao were closely related to the behaviour of villagers working and resting without protection in the mountains near the village [17]. This shows it is important to understand the spatial distribution of malaria at a finer geographic scale.

Three likely clusters of malaria cases identified with the time-space clustering were all found before June 2007. This showed that the incidence of malaria had significantly dropped within the whole study area since that time. Instead of natural factors such as region climate, this result is mostly likely contributed to powerful control measures, such as using insecticide-treated nets (ITNs), indoor residual spraying(IRS) and other methods of vector control, intermittent preventive treatment(IPT) for high-risk populations and health education.

SaTScan is a software that analyses spatial and temporal data using spatial, temporal, or space-time scan statistics. It is designed to perform geographical surveillance of disease, to detect spatial or space-time disease clusters, and to see if they are statistically significant; to test whether a disease is randomly distributed over space, over time or over space and time; to evaluate the statistical significance of disease cluster alarms; and to perform repeated time-periodic disease surveillance for early detection of disease outbreaks [12]. A comparative review of software packages for space-time disease surveillance found SaTScan as the most developed and robust software package for implementation in an automated cluster detection system [18]. SaTScan has been effectively used to analyse and characterize the spatial and temporal patterns of infectious diseases in recent years [6,8,19-23].

All the clusters detected in this study were circular. However, malaria might usually assume other cluster shape and more complicated clusters are more realistic in reality. This indicates the methodological limitation and cautions in results interpretation.

## Conclusions

This study analysed with the scan statistic the spatiotemporal characteristics of malaria variations of distribution at the village level within hyper-endemic areas in Hainan between 2005 and 2009. In summary, there was an obvious decreasing trend from 2005 to 2009 within the study area, and the distribution of malaria had significant spatial and temporal heterogeneities. This study could be helpful to prioritize resources assignment and investigation of malaria in endemic area for the disease control at a finer geographic scale. Further studies should be carried out to explain those spatial and temporal heterogeneities.

## Competing interests

The authors declare that they have no competing interests.

## Authors' contributions

LW designed the research, collected and analysed data and wrote the first draft of the manuscript. CL contributed to research execution, data analysis and drafted manuscript. ML contributed data collection and results interpretation. ZY, DH, SL, YW, CC, RJ and HS all advised on the design of the study, and the analysis of the results. All authors read and approved the final manuscript.
